# Challenges and Innovations in Postoperative Care for Acute Type A Aortic Dissection: The Role of Structured Surveillance and Virtual Wards

**DOI:** 10.31083/j.rcm2511389

**Published:** 2024-10-31

**Authors:** Robert Pruna-Guillen, Laerke Ghosh, Tara M. Mastracci, Vikas Kapil, Ana Lopez-Marco, Aung Oo, on behalf the of the rest of the aortic dissection surgical team at Barts Heart Centre (London, UK)

**Affiliations:** ^1^Department of Cardiothoracic Surgery, St Bartholomew's Hospital, EC1A 7BE London, UK; ^2^Barts Blood Pressure Centre of Excellence, Barts Heart Centre, St Bartholomew's Hospital, EC1A 7BE London, UK; ^3^William Harvey Research Institute, NIHR Barts Biomedical Research Centre, E1 4NS London, UK; ^4^Centre for Cardiovascular Medicine and Devices, Queen Mary University London, E1 4NS London, UK

**Keywords:** aortic dissection, Type A aortic dissection, postoperative management, guideline-directed imaging surveillance, virtual wards, blood pressure control

## Abstract

Acute Type A aortic dissection (ATAAD) represents a life-threatening medical emergency that requires emergent surgical repair. Despite improvement in surgical techniques and perioperative management, ATAAD remains associated with high early mortality and postoperative complications. A structured and individualized postoperative surveillance program is essential, not only for improving survival rates but also for identifying risk factors necessitating reintervention and enhancing the quality of life. Comprehensive postoperative care should address both medical monitoring and psychological support to meet the holistic needs of ATAAD survivors. In real-world settings adherence to guideline-directed imaging surveillance (GDIS) is poor, leading to underestimation of reintervention rates. A comprehensive aortic service should include GDIS, clinical assessments, cardiovascular risk management, and psychological support. Since August 2022, a virtual ward has been implemented in our department to facilitate remote monitoring, ensuring tight blood pressure control and early detection of complications.

Acute Type A aortic dissection (ATAAD) represents a life-threatening medical emergency that requires emergent surgical repair. Despite improvement in surgical techniques and perioperative management, ATAAD remains associated with high rates of early mortality and postoperative complications [1, 2, 3]. Therefore, effective postoperative management of ATAAD remains paramount for improving long-term outcomes and patient survival. For those who survive, a structured and individualized postoperative surveillance program is mandatory, as it contributes not only to improve survival rates but also to identify risk factors that could necessitate reintervention and to enhance the quality of life.

The physical and psychological impact of surviving ATAAD demand a holistic approach to postoperative care, this should include both medical monitoring and psychological support to address the comprehensive needs of patients recovering from ATAAD [4]. Outcomes of surgery for ATAAD extend beyond just mortality, morbidity and reoperation rates; therefore, quality of life scores should be reported too.

Historically, postoperative ATAAD follow up has been expressed with survival rate, evolution of residual dissected aortic segments, reinterventions and dissection related events [5, 6]. Multiple risk factors have been associated with aneurysmal dilatation and dissection-related events in postoperative ATAAD patients, such as young age, male sex, connective tissue disease, preoperative aortic diameter, limited distal aortic repair, presence of distal re-entry tears, distal anastomotic new entry tears (DANE), and patent false lumen, among others [7, 8, 9, 10]. Early identification of these risk factors should prompt more aggressive treatment strategies in the initial phase. Both American [11] and European [12] latest aortic guidelines recommend tailored surveillance programs for each patient. The 2022 American College of Cardiology/American Heart Association (ACC/AHA) Guideline for the Diagnosis and Management of Aortic Disease advises postoperative imaging with computed tomography (CT) Aortogram (or magnetic resonance imaging (MRI)) at 1, 6, and 12 months, and annually thereafter if stable [11]. However, adherence to these recommendations in real-world settings is poor, with less than 15% of patients receiving guideline-directed imaging surveillance (GDIS) in some cohorts​ [13]. Poor adherence to individualized follow-up is associated with worse survival [6, 14], this could suggest that reinterventions and dissection-related events are underestimated due to inadequate followed-up. In this scenario, the surgical community should reconsider using reintervention as an endpoint for aortic surveillance. A low reintervention rate does not necessarily indicate adequate outcomes; it may simply expose inadequate follow-up.

Strict blood pressure control is another cornerstone of postoperative management for patients who have undergone repair for ATAAD [15]. Effective blood pressure management has been strongly associated with increased survival rates and improved long-term outcomes by mitigating stress on the aortic wall and ensuring the stability of the surgical repair​ [16]. Regrettably, there has been no evidenced schedule or method for monitoring patients’ blood pressure after discharge, with many relying on their community health centres for follow-up care.

At our department in Barts Heart Centre in London, UK, we believe that an adequate life-long postoperative aortic follow-up is mandatory. A comprehensive aortic service, aimed at effective secondary prevention after repair of ATAAD, includes guideline-directed imaging surveillance, clinical assessments, management of cardiovascular risk factors with a focus on tight blood pressure control, and psychological support. Additionally, genetic testing is conducted in selected cases.

Since August 2022, we have implemented a virtual ward as a remote monitoring tool as part of patient aortic surveillance. Virtual ward models use digital solutions to deliver healthcare remotely and have gained increasing acceptance since the COVID-19 pandemic within the National Health Service (NHS)​ with multiple applications [17, 18]. ORTUS, developed by Ortus-iHealth (London, United Kingdom), is a virtual ward platform designed to enhance remote patient monitoring and management, particularly for cardiology and cardiothoracic surgery. This innovative system captures and tracks vital signs and patient-reported symptoms through connected devices like blood pressure monitors, which sync with the ORTUS app for real-time data access. Clinicians benefit from detailed dashboards that provide insights into patient health trends, enabling timely and prioritized care. It includes features for patient engagement, such as questionnaires, appointment booking, and multimedia consultation. According to previously published NHS experiences with virtual wards in arrhythmia clinics following catheter ablation [19], the use of ORTUS has significantly improved clinical outcomes. Specifically, there has been a 75% reduction in missed appointments and a 30% increase in clinical capacity. Patient satisfaction is notably high, with 87% of users reporting positive experiences. Additionally, the platform has greatly improved medication management, increasing the percentage of patients at optimal medication levels from 11% to 88%.

Among its benefits, the most important is establishing a direct communication channel between the patient and the specialized aortic team. This allows for strict blood pressure control and symptom monitoring, enabling the detection of symptomatic patients with early complications for timely treatment. Additionally, the virtual ward aims to reduce hospital visits and geographical exclusions, empower patients, increase treatment adherence, reduce local doctor visits, and facilitate early discharge. The main disadvantages are associated with patients who suffer from digital exclusion, such as the elderly or those experiencing social deprivation, and the allocation and dedication of clinician time.

From August 2022 to April 2024, 197 patients have been enrolled in our virtual ward, following acute aortic syndrome. Of these, 82% (163) have activated their account and 69% (139) have uploaded their blood pressure readings. Among these patients, 52 (26%) have required specialist antihypertensive referral to improve their blood pressure control. Enrolment involves that patients get nursing-delivered education about ATAAD implications and twice-weekly monitoring by the clinical team. Medication adjustments are based on antihypertensive guidelines and communicated through phone appointments and written correspondence, supervised by specialized antihypertensive cardiologists when required.

The aortic nurse plays a pivotal role in coordinating care, patient education, and ensuring adherence to follow-up protocols. Moreover, their involvement is important in monitoring patient progress, identifying early complications, and offering psychological support, thus contributing to improve patient outcomes and quality of life.

Effective postoperative management of ATAAD is essential for improving long-term outcomes and patient survival. Structured and individualized surveillance programs, strict blood pressure control, and comprehensive support systems, including psychological support, are key components of this management. Our experience at Barts Heart Centre demonstrates that innovative approaches, such as virtual wards, can effectively support postoperative care, enhancing patient monitoring and promoting adherence to treatment protocols.
